# Evaluation of Carbon Nanoparticle Suspension and Methylene Blue Localization for Preoperative Localization of Nonpalpable Breast Lesions: A Comparative Study

**DOI:** 10.3389/fsurg.2021.757694

**Published:** 2021-11-23

**Authors:** Yeqing Zhou, Yiran Liang, Jianshu Zhang, Yang Feng, Xiaoyan Li, Xiaoli Kong, Tingting Ma, Liyu Jiang, Qifeng Yang

**Affiliations:** ^1^Department of Breast Surgery, General Surgery, Qilu Hospital, Cheeloo College of Medicine, Shandong University, Jinan, China; ^2^Department of Breast Surgery, The Affiliated Yantai Yuhuangding Hospital of Qingdao University, Yantai, China; ^3^Pathology Tissue Bank, Qilu Hospital of Shandong University, Jinan, China; ^4^Research Institute of Breast Cancer, Shandong University, Jinan, China

**Keywords:** nonpalpable breast lesions, carbon nanoparticle suspension localization, methylene blue localization, breast cancer, preoperative localization

## Abstract

**Background:** The resection of nonpalpable breast lesions (NPBLs) largely depends on the preoperative localization technology. Although several techniques have been used for the guidance of NPBL resection, more comfortable and effective methods are needed. This aim of this study was to evaluate the use and feasibility of carbon nanoparticle suspension (CNS) and methylene blue (MB)-guided resection of NPBL, to introduce alternative techniques.

**Methods:** A total of 105 patients with 172 NPBLs detected by breast ultrasound were randomized to CNS localization (CNSL) group and MB localization (MBL) group. The injection times of the two groups were divided into 2, 4, 6, 12, 16, and 20 h before surgery. In this study, localization time, stained area, operation time, total resection volume (TRV), calculated resection ratio (CRR), and pathological diagnosis were assessed.

**Results:** All of the 172 lesions were finally confirmed benign. Dye persisted in all cases in the CNSL group (109/109, 100%), while that persisted in only 53 cases in the MBL group (53/63, 84.1%) (*P* < 0.001). There was a significant correlation between dyeing time and dyeing area in the MBL group (*r* = −0.767, *P* < 0.001); however, there was no significant correlation in the CNSL group (*r* = −0.154, *P* = 0.110). The operation time was 11.05 ± 3.40 min in the CNSL group and 13.48 ± 6.22 min in the MBL group (*P* < 0.001). The TRV was 2.51 ± 2.42 cm3 in the CNSL group and 3.69 ± 3.24 cm3 in the MBL group (*P* = 0.016). For CRR, the CNSL group was lower than the MBL group (7.62 ± 0.49 vs. 21.93 ± 78.00, *P* = 0.018). There is no dye remained on the skin in the MBL group; however, dye persisted in 12 patients (19.4%) in the CNSL group (*P* = 0.001).

**Conclusion:** Carbon nanoparticle suspension localization and MBL are technically applicable and clinically acceptable procedures for intraoperatively localizing NPBL. Moreover, given the advantages of CNSL compared to MBL, including the ability to perform this technique 5 days before operation and smaller resection volume, it seems to be a more attractive alternative to be used in intraoperative localization of NPBL.

## Introduction

With the advancements in mammographic screening procedures and imaging techniques, the detection rate of nonpalpable breast lesions (NPBLs) has increased significantly. Core needle biopsy (CNB) is a minimally invasive and effective modality to diagnose these lesions (1), with smaller surgical incision and less intraoperative blood loss. However, the existence of false-negative rate, underestimation of atypical ductal hyperplasia or Ductal Carcinoma *in situ* lesions, and excessive duct injury restrict its application to some extent (2, 3). Excisional biopsy is usually performed for these lesions, and accurate preoperative localization is needed to improve the detection rate of NPBL and reduce the operating time.

Various techniques have been used for localizing NPBL, such as wire-guided localization (WGL), intraoperative ultrasonographic imaging (IOUS), radio-guided occult lesion localization (ROLL), radioactive seed localization (RSL), and magnetically guided localization (MGL). However, these methods suffer from several disadvantages. WGL is the most commonly used technique to localize the lesions just before surgery (4). However, WGL is usually performed on the day of surgery, making the operation time more inflexible (5). Moreover, WGL can be associated with the patient discomfort, risks of wire migration or fracture, and interference with surgical approach (6–8). On the other hand, extra ultrasound knowledge and experience as well as prolonged operation time are needed for surgeons to perform IOUS (9). Moreover, the need of specific detection equipment and the radioactive materials as well as the coordination between radiologists and surgeons for ROLL, RSL, and MGL make them less flexible and limited for preoperative localization of NPBL (10). Therefore, a safe, low cost, and effective alternative is urgently needed.

The methylene blue (MB) and carbon nanoparticle suspension (CNS) has been widely used in sentinel lymph node (SLN) biopsy in breast cancer; however, their usefulness as adjuncts to the excision of NPBL remains to be evaluated. MB is a readily available and inexpensive dye, and previous studies have reported the use of MB in localizing NPBL (11). Although high detection rate of NPBL was achieved by MB, it is easy to diffuse in gland with rapid metabolism (12). Therefore, MB localization (MBL) needs to be performed within few hours before surgery. However, there is no universal standard for the preoperative injection time of MBL in existing reports. With the advancements in nanotechnology, a novel method of using CNS for preoperative localization of NPBL has been described (13). The safety of CNS has been proved in the operation of gastrointestinal cancer, thyroid cancer, and breast cancer (14, 15). Moreover, CNS is stable and uneasy to disperse within breast tissues; therefore, surgery can be delayed for days or even weeks. However, there are limited reports about the use of CNS for NPBLs.

In this study, we evaluate the usefulness of CNS localization (CNSL) and MBL-guided resection of NPBLs to provide fundamentals for the novel alternative methods.

## Materials and Methods

### Selection of Patients

In this prospective randomized clinical trial, 105 patients (172 lesions) were included during April 2018 to January 2020. All patients were confirmed to have NPBLs based on the results of breast ultrasound and then received surgery in Qilu Hospital of Shandong University, China. All patients were randomized to the CNSL group or the MBL group. Informed consents were obtained from all patients. This study was approved by the Ethical Committee of Qilu Hospital of Shandong University.

### Preoperative Localization and Surgery

**Figure 1:**
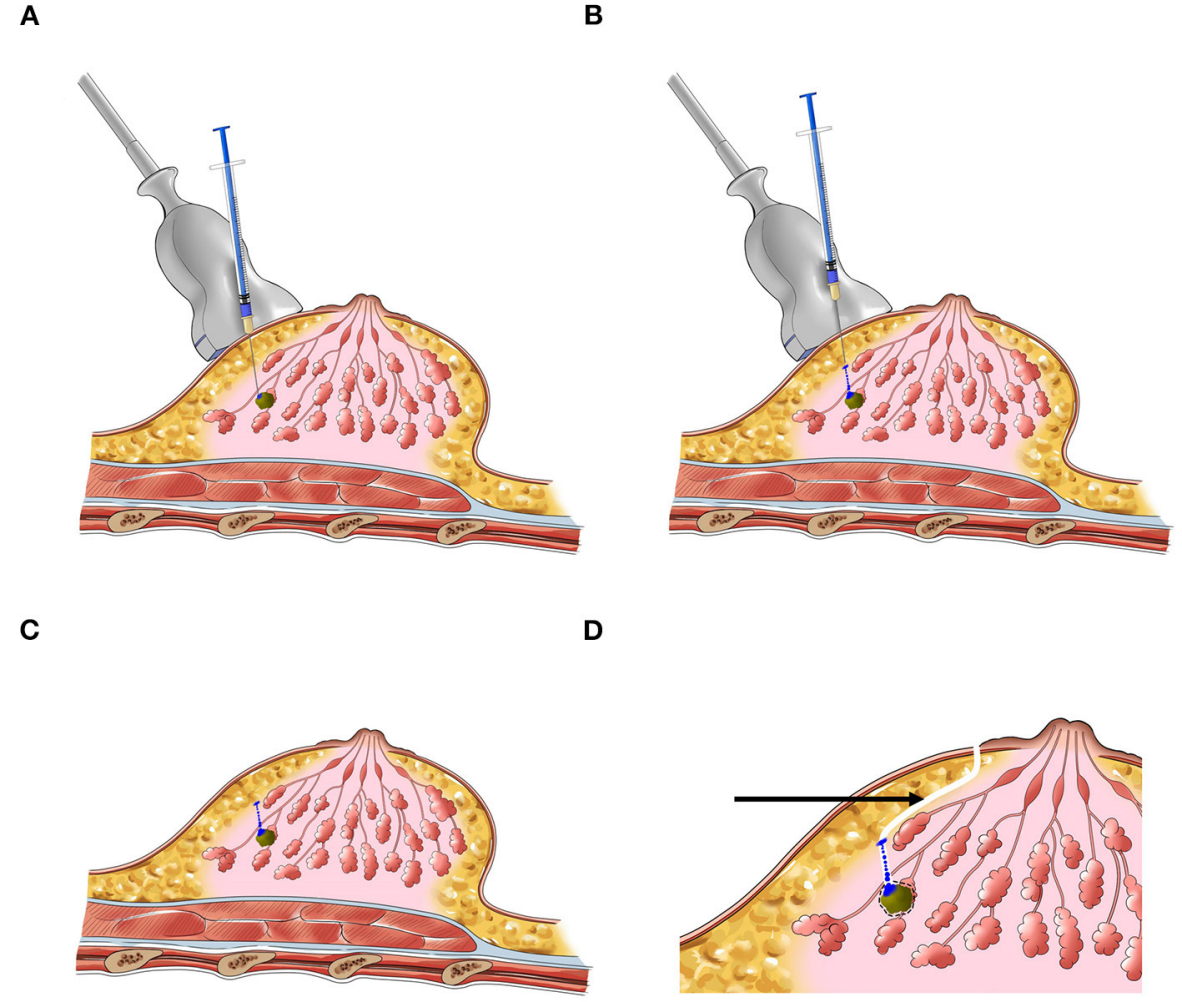
The schematic diagram of preoperative localization of NPBL. **(A–C)** Injection process. **(D)** Surgery pathway (the black arrow).

Before surgery, each lesion needed to be localized and marked on body surface under ultrasound, and the diameters of lesions were measured. Under the guidance of ultrasound, 0.02 ml of the CNS (Lummy Pharmaceutical Company, Chongqing, China) or MB (Jumpcan Pharmaceutical Company, Jiangsu, China) was injected into the gland from the deep to the shallow along the three sites of “lesion's surface–gland middle–gland surface,” and the needle was slowly pulled out while withdrawing the plunger of the syringe (Figure 1). The injection times of the two groups were divided into 2, 4, 6, 12, 16, and 20 h before surgery. Given the stability of the CNS, the injection time of the CNSL group was extended to 24, 48, and 72 h before surgery.

**Figure 2:**
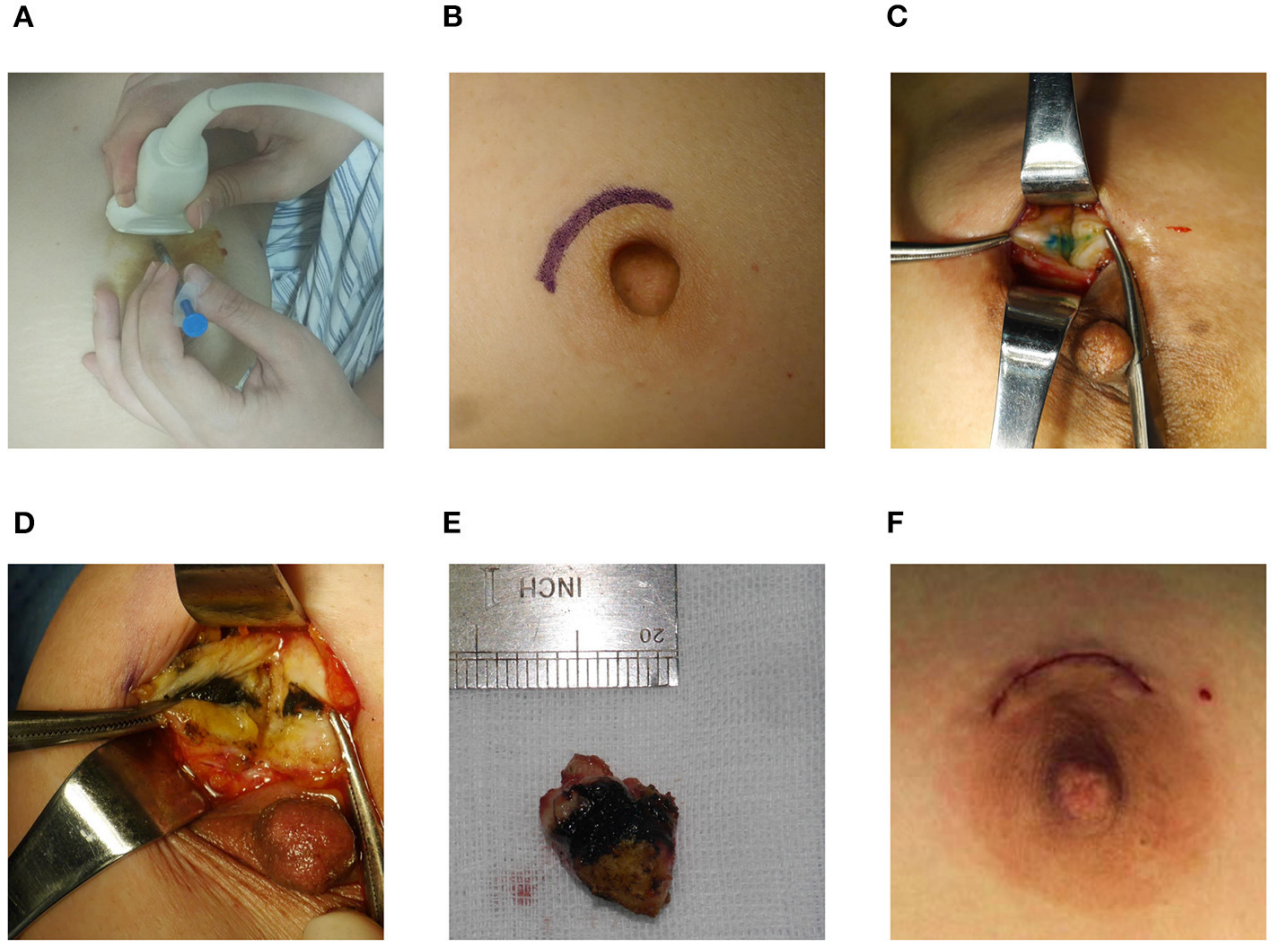
Surgical procedure. **(A)** Preoperative localization. **(B)** Periareolar incision. **(C,D)** Dyeing track. **(E)** Resection tissue. **(F)** Intradermal sutured incision.

All surgical procedures were performed by the same senior breast surgeon. Following the periareolar incision, the fat layer from the gland surface was dissociated to expose the dyeing site and the lesion was excised along the dyeing track later (Figure 2). The radii of dyeing diffusion and the dimensions of the resection tissues were measured. If there was no dyeing, the lesion could be excised following the surface marker. The participants first received breast lumpectomy, and the excised specimens were performed for the intraoperative pathological assessment. If the pathology result was benign, the surgical residual cavity was sutured. If the pathology result was malignant, the modified radical mastectomy was performed. All the removed specimens were sent for pathological examination.

### Evaluation Parameters

Both lesions and excised tissues could be considered as ellipsoids. The excised tissues volume could be indicated by the total resection volume (TRV), calculated by the formula: $\text{TRV}=\frac{\text{4}}{\text{3}}\text{π}\cdot \frac{\text{a}}{\text{2}}\cdot \frac{\text{b}}{\text{2}}\cdot \frac{\text{c}}{\text{2}}$ (a, b, and c were the excised tissue dimensions). For benign lesions, the lesion volume could be regarded as the optimal resection volume (ORV), $\text{ORV}=\frac{\text{4}}{\text{3}}\text{π }\cdot \;{\left(\frac{\text{d}}{\text{2}}\right)}^{3}$ (d was the diameter measured by pathological examination). For malignant tumors, previous studies (16, 17) have demonstrated that the OVR was calculated as lesion radii plus 1 cm margin of healthy breast tissue by the formula: $\text{ORV}=\frac{\text{4}}{\text{3}}\text{π }\cdot \;{\left(\frac{\text{d}}{\text{2}}+1\right)}^{3}.$ The calculated resection ratio (CRR) is expressed as the amount of excess breast tissue resected with the formula: CRR = TRV/ORV.

### Statistical Analysis

For the statistical analysis, SPSS 25.0 software package was used. Baseline patient characteristics and lesion features were compared between CNSL and MBL groups using the chi-square test. Student's *t*-test was used for the evaluation of statistical differences between two groups. The correlation was analyzed using Pearson's correlation analysis. *P* < 0.05 were considered statistically significant.

## Results

A total of 172 lesions were identified and preoperative dye localization was performed, including 109 lesions (63.4%) in the CNSL group and 63 lesions (36.6%) in the MBL group. The patient and lesion characteristics are listed in Tables 1 and 2, respectively. No allergic reaction or local bleeding was observed in two groups.

**Table 1:** Patient characteristics.

	**CNSL**	**MBL**	**Total**	***P*** **-value**
No. of patients	62	43	105	
**Age**				
<45	44	30	74 (70.5%)	0.532
≥45	18	13	31 (29.5%)	
**BMI**				
<24	36	24	60 (57.1%)	0.488
≥24	26	19	45 (42.9%)	
**No. of lesions**				
Solitary	35	27	62 (59.0%)	0.328
Multiple	27	16	43 (41.0%)	

*CNSL, carbon nanoparticle suspension localization; MBL, methylene blue localization; BMI, body mass index*.

**Table 2:** Lesion characteristics.

	**CNSL**	**MBL**	**Total**	***P*** **-value**
No. of lesions	109	63	172	
**Diameter (cm)**				
<1	78	41	119 (69.2%)	0.236
≥1	31	22	53 (30.8%)	
**BI-RADS**				
3	75	42	117 (68.0%)	0.450
4	34	21	55 (32.0%)	
**Lesion location**				
Left	47	33	80 (46.5%)	0.155
Right	62	30	92 (53.5%)	

*CNSL, carbon nanoparticle suspension localization; MBL, methylene blue localization*.

**Figure 3:**
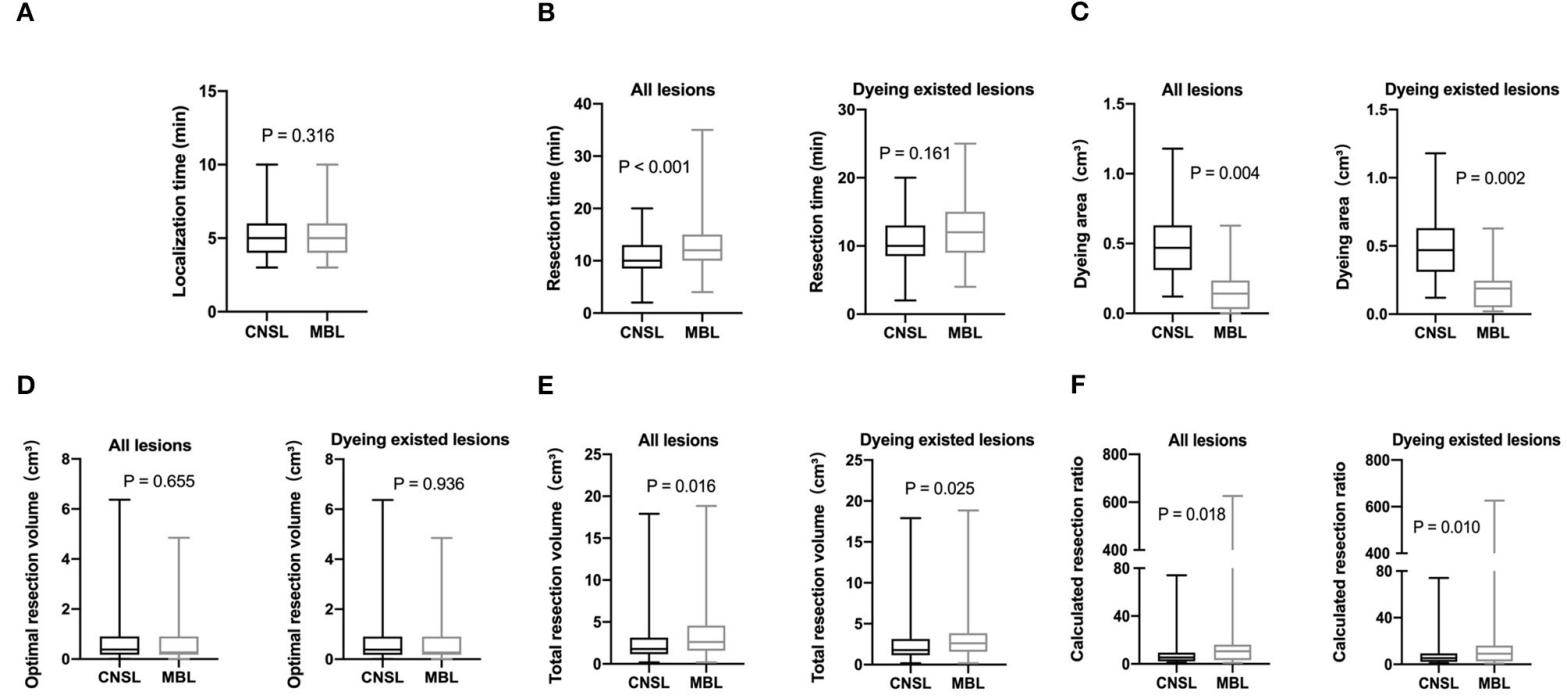
Clinical features of lesions localization. **(A)** Localization time (min). **(B)** Resection time (min). **(C)** Dyeing area (cm3). **(D)** Optimal resection volume (ORV) cm3. **(E)** Total resection volume (TRV) cm3. **(F)** Calculated resection ratio (CRR=TRV/ORV).

There was no statistical significance in the localization time between two groups (*P* = 0.316, Figure 3A). Significantly, the dyeing existed in all lesions of the CNSL group (109/109, 100%) and in 53 lesions (53/63, 84.1%) of the MBL group (*P* < 0.001, Table 3). Although the resection time of the CNSL group was shorter compared to the MBL group (*P* < 0.001, Figure 3B), the difference between the two groups with resection time was not statistically significant when excluded the dyeing disappeared cases (*P* = 0.161, Figure 3B). The mean dyeing area of the CNSL group was larger compared to that of the MBL group (Figure 3C). There was no statistical significance on the ORV between the CNSL group and the MBL group (Figure 3D). Moreover, the TRV and CRR in the CNSL group was lower compared to those of the MBL group in all lesions (*P* = 0.016 and *P* = 0.018, respectively, Figures 3E,F), and the difference between the two groups was also statistically significant when excluded the dyeing disappeared cases (*P* = 0.025 and *P* = 0.010, respectively, Figures 3E,F). The detailed results are presented in Table 3.

**Table 3:** Clinical features of lesions localization.

	**CNSL**	**MBL**	***P*** **-value^a^**
Localization time(min)	5.34 ± 1.71	5.37 ± 1.53	0.316
**Dyeing outcome**			
existed	109	53	<0.001^b^
disappeared	0	10	
**Resection time(min)**			
All lesions	11.05 ± 3.40	13.48 ± 6.22	<0.001
Dyeing existed	11.05 ± 3.40	11.91 ± 4.34	0.161
**Dyeing area(cm**3**)**			
All lesions	0.50 ± 0.22	0.16 ± 0.15	0.004
Dyeing existed	0.50 ± 0.22	0.19 ± 0.15	0.002
**TRV(cm**3**)**			
All lesions	2.51 ± 2.42	3.69 ± 3.24	0.016
haiDyeing existed	2.51 ± 2.42	3.50 ± 3.37	0.025
**ORV(cm**3**)**			
All lesions	0.66 ± 0.82	0.61 ± 0.78	0.655
Dyeing existed	0.66 ± 0.82	0.64 ± 0.84	0.936
**CRR**			
All lesions	7.62 ± 0.49	21.93 ± 78.00	0.018
Dyeing existed	7.62 ± 9.49	23.31 ± 85.02	0.010

*CNSL, carbon nanoparticle suspension localization; MBL, methylene blue localization*.

a *Student's t test*.

b *Chi-square test*.

**Figure 4:**
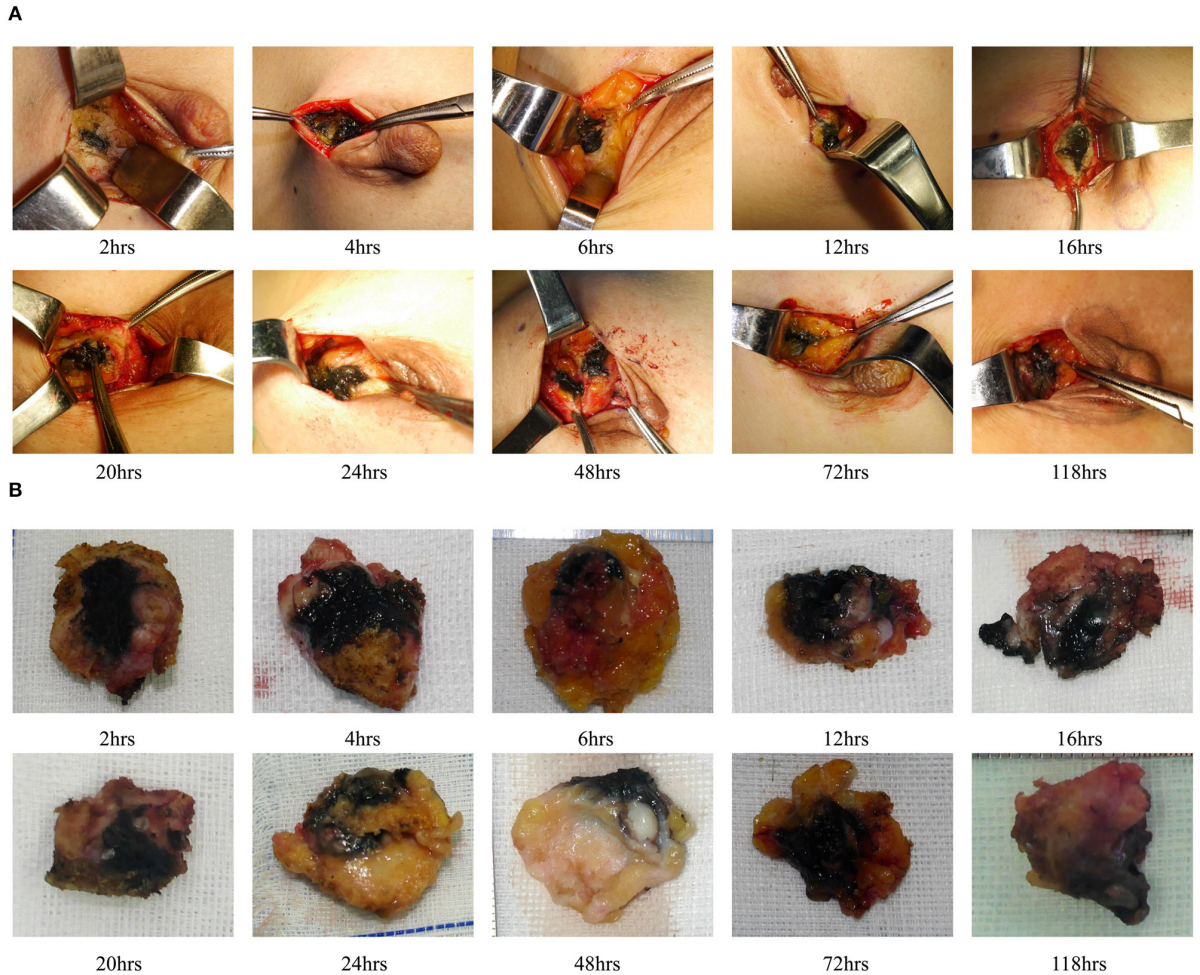
Dyeing in CNSL group. **(A)** Dyeing track. **(B)** Resection tissues.

**Figure 5:**
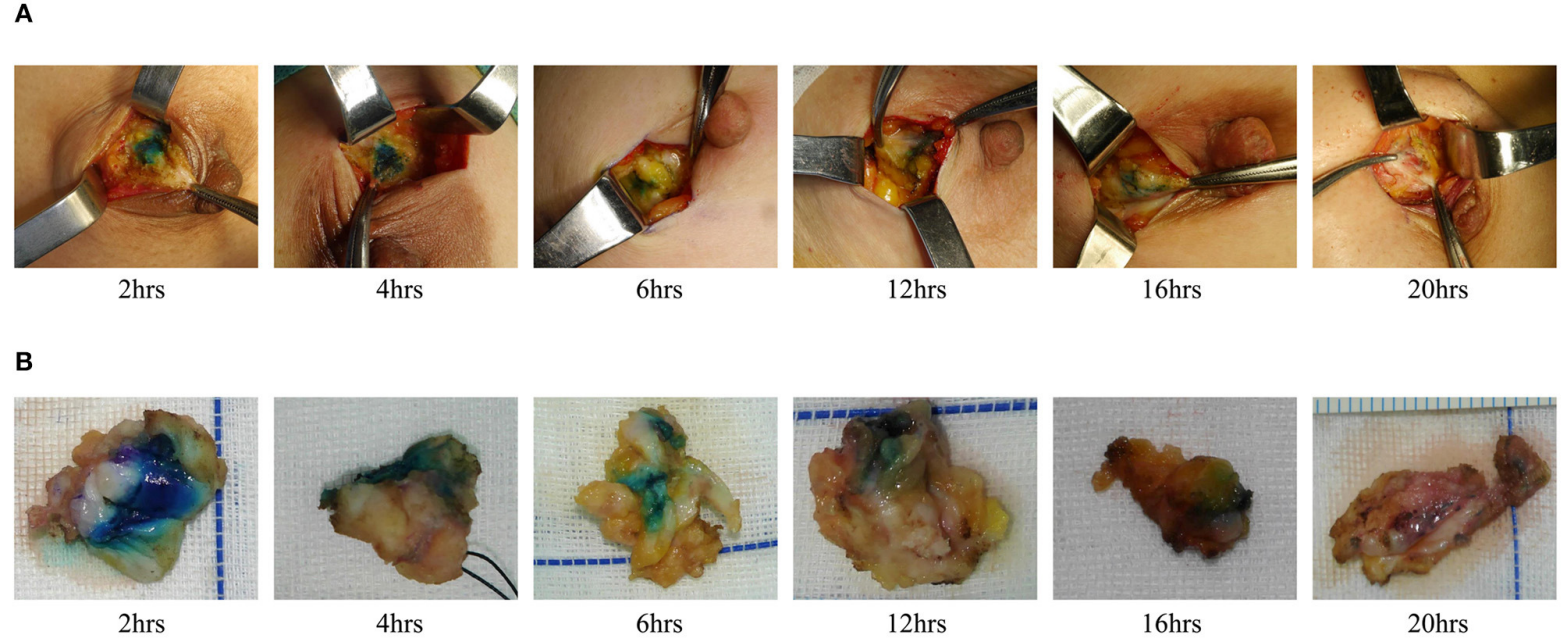
Dyeing in MBL group. **(A)** Dyeing track. **(B)** Resection tissues.

**Figure 6:**
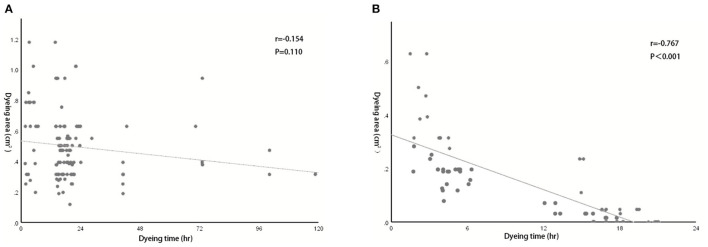
Correlation between dyeing time and dyeing area. **(A)** Correlation in the CNSL group (*r* = −0.154, *P* = 0.110). **(B)** Correlation in the MBL group (*r* = −0.767, *P* < 0.001).

We also evaluated the effect of dyeing time on the dyeing areas and dyeing intensity in two groups. The longest dyeing time in the CNSL group was 118 h (about 5 days). With the increase in dyeing time, the dyeing area and dyeing intensity of the CNSL group did not change significantly (Figure 4). In the MBL group, both dyeing area and intensity decreased with the prolonging of dying time (range from 2 to 20 h) (Figure 5). There was no significant correlation between the dyeing time and dyeing area in the CNSL group (*r* = −0.154, *P* = 0.110) (Figure 6A); however, the dyeing area was negatively associated with the dyeing time in the MBL group (*r* = −0.767, *P* < 0.001) (Figure 6B).

The pathologic diagnosis demonstrated that all of the 172 lesions were benign. Ninety-four lesions in the CNSL group and 53 lesions in the MBL group were diagnosed as fibroadenoma. Eleven and two cases of intraductal papilloma were identified in the CNSL and MBL groups, respectively. There were two ductal ectasia cases in each group. Three cases were mammary ductal ectasia (one in CNSL and two in MBL), and five cases were sclerosing adenosis (one in CNSL and four in MBL). Significantly, there were no positive margins and re-resection among all the 172 cases.

During the time of follow-up (2–18 months), patients had no complication occurred, such as incision infection, nipple, or areola ischemia. The NPBL recurred in nine patients (14.5%) in the CNSL group and eight patients in the MB group (18.6%) (*P* = 0.537). A total of 16 patients developed local collapse (11 in CNSL and five in MBL, *P* = 0.408). Thirteen patients (21.0%) had obvious scar in the CNSL group and 3 (7.0%) in the MBL group. There was no dye remained on the skin in the MBL group; however, dye remained in 12 patients (19.4%) in the CNSL group (*P* = 0.001). The detailed results are listed in Table 4.

**Table 4:** Postoperative data during the time of follow-up.

	**CNSL**	**MBL**	***P*** **-value^a^**
No. of follow-up	53 (85.5%)	36 (83.7%)	0.805
Recurrence	9 (14.5%)	8 (18.6%)	0.537
Local collapse	11 (17.7%)	5 (11.6%)	0.408
Obvious scar	13 (21.0%)	3 (7.0%)	0.089
Dyeing residual	12 (19.4%)	0	0.001
Satisfaction	53 (100.0%)	36 (100.0%)	

*CNSL, carbon nanoparticle suspension localization; MBL, methylene blue localization*.

a *Chi-square test*.

## Discussion

With the increasing number of NPBLs detected by advanced mammography screening programs, the need for rapid and precise localization is highlighted. Among the techniques utilized for the preoperative localization of NPBL, WGL is the most widely used method. However, there are several downsides of this method (18), such as the high risk of positive margin, wire migration, or breakage. Although IOUS could reduce the positive margin rate, ultrasound knowledge and experience were required for the surgeons (19, 20). Moreover, ROLL and RSL have been proposed as an alternative to WGL, which are performed by a radiologist immediately before the surgical procedure. Previous study reported lower rates of positive margin and re-excision in ROLL and RSL compared to WGL (21); however, the radioactive contamination and requirement of additional detection equipment limited their application (5). Recently, MGL has been reported to be useful in the detection and excision of NPBLs. However, there are several drawbacks for MGL (22, 23), such as the need for detection equipment, the limited use of metal instruments during surgery, and limited detection depth.

Methylene blue and CNS are two excellent and safety dyes for clinical use. Although most of the reports for CNS and MB applied are about the intraoperative identification of SLN, the techniques are feasible as guidance to the localization of NPBLs with theoretical and practical advantages. In 1976, Dietler et al. described the use of MB in the localization of NPBLs, which showed high accuracy, patient acceptance, and smaller size of the biopsy specimen (24). Subsequently, Tang et al. further demonstrated the safety, simpleness, and high diagnostic accuracy of MB in the localization and excision of NPBLs (11). Another study compared the combined use of ROLL and MB to WGL in resection of NPBLs (25) and revealed that the combined method could provide precise localization of NPBLs, leading to a clearer margin and a smaller specimen size. In this study, we summarized our experience using MB in 63 cases and showed high effectiveness in the intraoperative localization of NPBLs with less discomfort compared to traditional techniques. CNS, as a novel type of carbon dye, is safer and uneasy to precipitate. Previous study demonstrated the feasibility of CNSL used in breast-conserving surgery (26), which used the multidirection localization of margin as guidance for the excision of nonpalpable breast cancer. In our study, we directly located NPBLs rather than the margin, which facilitated the visualization of the whole process of excising the lesion during surgery and no re-excision case exists in two groups. Therefore, our results demonstrated that the use of MB or CNS alone could achieve complete resection of NPBL.

One priority concern about MB or CNS is the possibility of diffusion of the dyes, which might make the excision of lesions difficult if surgery is not performed in time after injection. In the MBL group, the dyeing time ranged from 2 to 20 h, and both dyeing area and staining intensity decreased as the staining time increased. We also identified a significant negative correlation between the dyeing time and dyeing area in the MBL group (*r* = −0.767, *P* < 0.001). In our study, when the dyeing time was within 6 h, an obvious blue area could be observed during the operation (radius of dyeing, 0.7 cm); when dyeing between 12 and 16 h, the staining area was significantly reduced (radius of dyeing, 0.3 cm); significantly, when dyeing between 16 and 20 h, the remaining dyeing was linear or dot-like or completely disappeared. Therefore, the optimal injection time before surgery was within 6 h for MBL, which means surgery should be performed on the same day. In terms of CNS, Jiang et al. (26) reported one case that CNS still existed 14 days after injection. In our study, the dyeing time of CNSL ranged from 2 to 118 h, and dyes existed in all cases in the CNSL group. With the increase in dyeing time, the dyeing area and dyeing intensity of the CNSL group hardly diminished. This flexibility in the injection time of CNS provides a clear advantage of this technique over MBL by reducing the time pressure on the operating rooms, thereby enabling better resource management on the day of surgery.

It has been reported that 15–20% of NPBL are malignant (27), however, all the 172 lesions were confirmed benign tumors by final pathology in the present study. Benign breast tumors (BBTs) are more common than malignant breast cancer (28) and may undergo malignant transformation. For patients with pain or anxiety and fertile woman, surgical resection is the main treatment of BBTs (29). BBTs resection through periareolar incision can not only resect the tumor thoroughly but also ameliorate the cosmetic problem (28). However, it has been previously reported that due to relatively limited exposure, the periareolar incision is difficult to accomplish and requires more time when the tumor is deep or locates in the breast edge (30, 31). In the MBL group, the mean resection time of 10 cases with no dyeing was 21.8 ± 8.16 min, which was almost double of the dyeing existed cases (11.91 ± 4.34 min). Our results also revealed that the resection time in the CNSL group was shorter than in the MBL group (11.05 ± 3.40 vs. 13.48 ± 6.22 min, *P* < 0.001). However, when excluded the dyeing disappeared cases, the difference between the two groups was not statistically significant (*P* = 0.161). Therefore, the result indicated that effective localization with dyes could shorten the operation time using periareolar incision.

In this study, both CNSL and MBL help to accomplish the complete resection of 172 lesions, indicating that the dye localization was a feasible and effective auxiliary technique in the resection of NPBL. Complete resection of NPBL while avoiding excessive resection of normal tissue is significant for cosmetic outcomes (32). Our results revealed that the TRV and CRR of the CNSL group were significantly lower compared to those of the MBL group. One possible explanation is that the dyeing area of MB decreases as time goes on, and the smaller dyeing range reduces the accuracy of localization, resulting in excessive resection of normal tissue. Compared with MBL, the CNSL not only achieves complete resection, but also reduces extra resection of normal tissues to improve cosmetic outcomes. Moreover, dye localization has less patient discomfort than other localization techniques. Previous studies have shown that the risk of allergic reactions ranges from 1 to 2% with MB (33). In addition, there have been a few cases about skin necrosis and fat necrosis following the injection of different dyes (34, 35). Up to the present moment, there have been no such complications found in this study. During the follow-up (2–18 months), 12 patients of the CNSL group have residual carbon marking of the skin at the injection site, affecting the appearance. No patient in the MBL group has residual dyeing. Moreover, no toxic and side effect was identified in the two groups, which further proved the safety of these two methods. CNS is produced through advanced nanotechnology and had applied for multiple patents. Therefore, another advantage of MB is that the price is much lower than that of the CNS due to the complex production process of CNS, making MB more favorable in financially restricted patients and health-care systems. Although the price of CNS is still within an affordable range for most patients, it would be more feasible for people if the price could be reduced with the wider use of CNS and the development of technologies.

There are several benefits of MBL and CNSL. The most significant advantage is that they could improve the flexibility of surgery time without losing accuracy. These techniques also provide direct visualization of the lesions during surgery, leading to decreased possibility of positive margins and less unnecessary sacrifice of healthy breast tissue. However, this study also had several limitations. First, limited by objective factors such as operating room arrangement, there is a lack of cases of dyeing between 8 and 10 h. Second, early unskilled operation of dye localization might lead to the occurrence of skin residual dyeing.

## Conclusion

In conclusion, CNSL and MBL are safe, simple, and effective method for the localization of NPBL with less discomfort and complications. For financially restricted patients and health-care systems, MBL is a lower-cost method and should be performed within 6 h before surgery. CNSL can be injected 5 days before surgery, which makes the operation time more flexible. In comparison with MBL, the CNSL can more effectively avoid excessive resection volume while ensuring complete resection. Therefore, CNSL and MBL are attractive alternatives for the localization of NPBL, although more studies are needed to further evaluate their feasibility in a larger cohort.

## Data Availability Statement

The raw data supporting the conclusions of this article will be made available by the authors, without undue reservation.

## Ethics Statement

The studies involving human participants were reviewed and approved by Ethical Committee of Qilu Hospital of Shandong University. The patients/participants provided their written informed consent to participate in this study. Written informed consent was obtained from the individual(s) for the publication of any potentially identifiable images or data included in this article.

## Author Contributions

YZ, YL, and QY planned and designed the study. XL, XK, TM, LJ, and QY performed the operation. YZ, YL, JZ, and YF collected data and performed statistical analysis. YZ, YL, JZ, and QY wrote and revised the manuscript. All authors reviewed and approved the final manuscript.

## Funding

This work was supported by National Key Research and Development Program (No. 2020YFA0712400), Special Foundation for Taishan Scholars (No. ts20190971), National Natural Science Foundation of China (No. 81874119; No. 82072912), Special Support Plan for National High Level Talents (Ten Thousand Talents Program W01020103), National Key Research and Development Program (No. 2018YFC0114705), Foundation from Clinical Research Center of Shandong University (No.2020SDUCRCA015), and Qilu Hospital Clinical New Technology Developing Foundation (No. 2018-7; No. 2019-3).

## Conflict of Interest

The authors declare that the research was conducted in the absence of any commercial or financial relationships that could be construed as a potential conflict of interest.

## Publisher's Note

All claims expressed in this article are solely those of the authors and do not necessarily represent those of their affiliated organizations, or those of the publisher, the editors and the reviewers. Any product that may be evaluated in this article, or claim that may be made by its manufacturer, is not guaranteed or endorsed by the publisher.
